# A Novel Chemometric Method for the Prediction of Human Oral Bioavailability

**DOI:** 10.3390/ijms13066964

**Published:** 2012-06-07

**Authors:** Xue Xu, Wuxia Zhang, Chao Huang, Yan Li, Hua Yu, Yonghua Wang, Jinyou Duan, Yang Ling

**Affiliations:** 1 College of Science, Northwest A & F University, Yangling 712100, China; E-Mails: xu_xue_1986@163.com (X.X.); lpeng@nwsuaf.edu.cn (W.Z.); 2 College of Life Science, Northwest A & F University, Yangling 712100, China; E-Mails: fishery18@163.com (C.H.); yuhua200886@yahoo.com.cn (H.Y.); 3 School of Chemical Engineering, Dalian University of Technology, Dalian 116024, China; E-Mail: adinalee@163.com; 4 Laboratory of Pharmaceutical Resource Discovery, Dalian Institute of Chemical Physics, Chinese Academy of Sciences, Dalian 116023, China; E-Mail: yling@dicp.ac.cn

**Keywords:** oral bioavailability, quantitative structure activity relationship, cytochrome P4503A4 and P4502D6, P-glycoprotein

## Abstract

Orally administered drugs must overcome several barriers before reaching their target site. Such barriers depend largely upon specific membrane transport systems and intracellular drug-metabolizing enzymes. For the first time, the P-glycoprotein (P-gp) and cytochrome P450s, the main line of defense by limiting the oral bioavailability (OB) of drugs, were brought into construction of QSAR modeling for human OB based on 805 structurally diverse drug and drug-like molecules. The linear (multiple linear regression: MLR, and partial least squares regression: PLS) and nonlinear (support-vector machine regression: SVR) methods are used to construct the models with their predictivity verified with five-fold cross-validation and independent external tests. The performance of SVR is slightly better than that of MLR and PLS, as indicated by its determination coefficient (*R*^2^) of 0.80 and standard error of estimate (SEE) of 0.31 for test sets. For the MLR and PLS, they are relatively weak, showing prediction abilities of 0.60 and 0.64 for the training set with SEE of 0.40 and 0.31, respectively. Our study indicates that the MLR, PLS and SVR-based *in silico* models have good potential in facilitating the prediction of oral bioavailability and can be applied in future drug design.

## 1. Introduction

A large number of compounds emerging from combinatorial chemistry and high throughput medicinal chemistry programs have increased the demand for new compounds that need to be screened in a wide range of biological assays [1]. It has been reported that 95% of lead compounds fail in the developmental stages, and 50% of these failures are induced by unfavorable absorption, distribution, metabolism, and excretion (ADME) properties [2,3]. Since the predominant and most convenient way to deliver drugs to the systemic circulation for patients is the oral route, the good oral bioavailability (OB) of a new drug candidate is undoubtedly one of the most important pharmacokinetic parameters along with ADME properties.

The OB is defined as “the rate and extent to which the active ingredient or active moiety is absorbed from a drug product and becomes available at the site of action” by FDA [4]. A low and highly variable bioavailability is the main reason for stopping further development of the drug candidates. In fact, in recent years, multiple large-scale experiments drugs candidates have been conducted to assess the OB values of molecules, but they are labor-intensive and time-consuming. Therefore, developing a reliable and efficient *in silico* method that can predict human OB is compelling [5,6], both in the early stage of drug discovery to select the most promising compounds for further optimization and in the later stage to identify final candidates for further clinical development.

Lipinski’s “Rule of Five”, which could be qualitatively used to predict the absorption and permeability of drug molecules, has so far been the primary guide to predicting OB [7]. Since then, numerous classification and regression models for the predictions of OB were proposed by applying various statistical and machine-leaning computational approaches [8,9]. However, most of these models cannot demonstrate satisfactory predictions for the bioavailability. In 2000, Andrews and co-workers constructed a regression model to predict OB based on a dataset of 591 molecules by employing 85 structural descriptors [8]. Compared to Lipinski’s “Rule of Five”, the false negative rate was reduced from 5% to 3%, and the false positive rate decreased from 78% to 53%. But it should be noted that this model is not very good considering the high rate of false positives and the 85 descriptors used, which would cause an overfitting problem. In the same year, Yoshida *et al*. established a classification model for predicting OB with 15 structural descriptors, in which three descriptors were closely related to distribution coefficients [9]. However, this model can only perform a correct accuracy of 60% for the test compounds, leading to the difficulty to construct highly reliable models.

It is a significant milestone that Hou and his coworkers have built a publicly available and reliable source for OB in 2007 [10]. Then, many new models based on the large and reliable database were proposed [11,12]. In 2008, Wang *et al*. showed that no good prediction model of general validity could be obtained with the full dataset of 772 compounds, and instead, have built four better models by manually selecting the compounds characteristic with similar structures or pharmacological activities [11]. However, the number of each subset is too small to cover all the 772 compounds. Subsequently, Ma and co-workers proposed a prediction model of OB using GA-CG-SVM, which gained receivable overall classification accuracy (~80%), but unreasonable prediction accuracy for the low-bioavailability class (~25%) [12]. In 2011, Tian *et al*. constructed multiple linear regression (MLR) models for OB based on molecular properties and structural fingerprints by employing the genetic function approximation (GFA) technique, but their prediction abilities were relatively low [13]. In short, no reliable prediction model for OB has been developed based on the simple descriptors so far.

Indeed, the human bioavailability involves a complex biological and physiological process that is influenced by various factors, including gastrointestinal transition and absorption, intestinal membrane permeation, and intestinal/hepatic first-pass metabolism [14]. P-glycoprotein (P-gp), as the most studied ATP-dependent efflux protein, enables to drive compounds from inside the cell back into the intestinal lumen, and thereby prevents their absorption into blood [15]. Such protein is present on the intestinal brush border, in close proximity to the main cytochrome P450 (CYP) isoenzymes responsible for the majority of intestinal drug metabolism, CYP3A4 and CYP2D6 [16]. The CYP system is the most important family of enzymes that carry out oxidation of drugs and xenobiotics, which contributes significantly to the first-pass metabolism of many drugs such as cyclosporine, midazolam, and verapami. Indeed, substrates for P-gp and CYP3A have great overlap covering diverse therapeutic indications and a broad range of molecular structures, which indicates that the two proteins act synergistically in preventing drugs from crossing the intestinal barrier [17]. Therefore, it is reasonable to believe that in combination with P-gp, the CYP enzymes are critical factors in determining the bioavailability of drugs.

It is frequently stated that the ideal model system for OB of drugs should be physiologically and sufficiently reflective of the specific biological barrier of interest in humans [18]. This emphasizes that the knowledge of the metabolism and efflux at the intestinal mucosal level is of particular importance. Thus in this work, to compensate for the lack of key information on the physiological and biochemical processes for the prediction of oral drug bioavailability, and to improve the reliability and efficiency of OB models, we have developed a novel chemometric method by integrating the properties of P-gp-mediated efflux and metabolism by P450. On the basis of a large-scale OB (%F, the amount of drug that reaches the systemic circulation after absorption and first pass clearance) database, the relationships between %F with the well-used molecular properties have been elucidated and the reliable prediction models for OB were constructed, which can be used as rapid screening filters for candidate drugs.

## 2. Materials and Methods

### 2.1. Dataset Construction

805 structurally diverse drug and drug-like molecules and their OB values (%F) in human were obtained from the bioavailability database [19]. The dataset encompassed a broad range of chemical substances. For the compounds with different bioavailability values, their average values were employed to reduce bias in the experiments. In this work, to guarantee the linear distribution of the biological data, all the OB values were transformed into the common logarithm of log (oral bioavailability) (logB). The structures of the components were downloaded from Chemical Book Database [20], or generated by ISIS Draw 2.5 (MDL Information Systems, Inc.), and then were optimized by Sybyl 6.9. The Sybyl parameters were set similarly to those in our previous work [21]. All the chemicals were saved as mol2 format for further analysis.

### 2.2. Molecular Descriptors

Construction of the models for OB firstly depends on the generation of molecular descriptors, which can be calculated directly from the structure of any particular molecule by simply using various molecular modeling tools. Dragon descriptors have been successfully used for quantitatively representing the structural and physicochemical features of a molecule [22–24]. In the present work, a total of 1536 molecular descriptors were calculated using dragon professional 5.4-2006 [25], including constitutional descriptors, topological descriptors, walk and path counts, connectivity indices, information indices, 2D autocorrelations, edge adjacency indices, Burden eigenvalues, topological charge indices, eigenvalue-based indices, Randic molecular profiles, geometrical descriptors, RDF descriptors, 3D-MoRSE descriptors, WHIM descriptors, GETAWAY descriptors, functional group counts, atom-centered fragments, charge descriptors and molecular properties. Constitutional descriptors are related to the number of atoms and bonds in the molecule. Topological descriptors are a special class of descriptors that do not rely on a three-dimensional model, including valence and no valence molecular connectivity indices calculated from the hydrogen suppressed formula of the molecule, encoding information about the size, composition, and the degree of branching of a molecule. Autocorrelation descriptors constitute a set of molecular descriptors derived from a conceptual dissection of the molecular topology and taking into account chemical information contained in the atomic weightings and structural information by specified weights of the molecule atoms. Radial Distribution Function (RDF) descriptors do not depend on the molecular size and take into account the 3D arrangement of the atoms without ambiguities, thus being applicable to a large number of molecules with great structural variance and being a characteristic common to all of them. Formally, the RDF of an ensemble of atoms can be interpreted as the probability distribution of finding an atom in spherical volume of radius R. The geometrical descriptors describe the size of the molecule and require 3D-coordinates of the atoms in the given molecule.

### 2.3. Database Division

All the 805 compounds were divided into several statistical subsets by using the Self-consistent method. First, the geometry-based algorithm [26] was applied to identify the binding modes of molecules with the metabolizing enzymes CYP3A4 and CYP2D6 and the efflux protein P-gp. The crystal structures of human CYP3A4 (PDB code: 3NXU) and CYP2D6 (PDB code: 2F9Q) were retrieved from RCSB Protein Data Bank [27]. Due to unavailability of the X-ray structure of human P-gp, a homology modeling for the protein was performed [28]. The template protein employed here was mouse P-gp (PDB code: 3G60 chain A [9]) which exhibited a high resolution (4.40 Å). This method employed three-dimensional transformations driven by local feature matching, and spatial pattern detection techniques such as the geometric hashing and pose clustering, to yield good molecular shape complementarity with high efficiency. After the fast transformational search, the best geometric fit obtained the highest scores (~5000), while the low scores (~500) exhibited poor matches. For the complexes in our work, the Clustering RMSD was 2.5 Å. The five lowest-binding energy matches for each complex were selected and analyzed visually.

Secondly, the iterative self-consistent approach was used for setting boundary for the subsets [29]. This method applied iterative steps to identify the maximum spatial distribution of molecules on the basis of their features, and estimate the statistical parameters of the spatial processes. In other words, the subsets are divided based on the correlation between the values of neighboring molecules. In the present work, the 805 molecules were first ranked by their binding affinities in descending order. Based on the ranking of binding affinities, one subset of the compounds with random number was selected at each of the end position, and then the correlations between the bioavailability of the molecules and their corresponding binding affinities were evaluated, respectively (step 1). The obtained determination coefficients (*R*^2^) were employed as a judgment for the boundary setting of molecules. The feature similarity of neighboring molecules was estimated to probe the maximum spatial gap:

(1) $$\underset{n}{\text{arg}\;\text{min}}\sum_{i=1}^{n}(\sum_{j=1}^{r_{i}}\sum_{k=1}^{r_{i}}\sqrt{{\left(x_{j}-x_{k}\right)}^{2}}/r_{i}(r_{i}-1))/n$$

where *n* is the number of subset, *r_i_* is sample number of subset *i*, and *x_j_*, *x_k_* are feature descriptor vectors for compound *j*, *k* of subset *i*.

Finally, steps 1 and 2 were repeated until the reliable *R*^2^ values were obtained for each subset.

### 2.4. Design of Training and Test Sets

The compounds in each subset were split into training and independent validation sets based on their distribution in the chemical space as defined by Self-organizing map (SOM). SOM is a type of artificial neural network that is trained using unsupervised learning to produce a low-dimensional representation of the input space of the training samples [30]. In the SOM, the procedure for placing the vector from data space onto the map is to find the neuron with the closest weight vector to the vector taken from data space and to assign the map coordinates of this neuron to the vector. A formal rule for the selection of the winner (out) is based on the Euclidean distance between a vector (*x*) and a weight (*w*).

(2) $${\text{out}}_{\text{sc}}\leftarrow \text{min}\left[\sum_{i=1}^{m}{\left(x_{si}-w_{ji}\right)}^{2}\right]$$

Then the weight of the winner is corrected to decrease this distance. In such a process, the SOM works as a clustering diagram grouping similar inputs from the input space into similar neurons of the output space.

The optimal 10 × 10, 7 × 6, 8 × 8, 8 × 8 node architectures were chosen to map objects into 100, 42, 64, 64 positions for Set 1, Set 2, Set 3 and Set 4, respectively. Similar compounds were clustered into the same position (*x*, *y* coordinate in a SOM). Only one part of a representative object from each position in the SOM map was chosen for the training set, respecting the original proportion and the predefined 4:1 ratio between the training and test objects. For each subset, the obtained training sets including 156, 122, 180 and 197 compounds were applied for the development of the modeling system, and the rest groups of 36, 27, 44 and 43 compounds as the independent evaluation set were used for the assessment of the system, respectively. The simulations were carried out using an internally developed C-language program.

### 2.5. MLR

As one of the most widely used methods for forecasting, MLR attempts to model the relationship between two or more explanatory variables and a response variable by fitting a linear equation to the observed data [31]. By interpreting the descriptors in the regression models it is possible to gain some insight into those factors that are likely to govern the OB of the compounds, which is very useful in the design of new drugs or to screen drug-like compounds starting only from the molecular graph. Generally, the more the parameters are used in regression equation the lower the value of residual sum of squares and the higher the value of explained sum of squares will be. However, models containing more correlating parameters may suffer from the defect of collinearity and containing inferior variables and omitting important ones, which will make the parameters’ estimation based on traditional methods not satisfactory. Therefore, considering the large number of molecular descriptors we used, the stepwise process is applied to choose the best combination of descriptors automatically and construct the multiple regression models with the highest statistical significance.

In this work, those variables with zero values (> 80%) were eliminated, with remaining molecular descriptors further selected by the stepwise method in MLR process. The Stepwise variable entry and removal examines the variables in the block at each step (criteria: probability of *F* to enter ≤ 0.05, probability of *F* to remove ≥ 0.10).

### 2.6. Partial Least Squares Analysis (PLS)

PLS, known as Projection to Latent Structures, is a powerful statistical method that can easily cope with a large number of correlated descriptors by projecting them into several orthogonal latent variables [32]. Being a component-based structural equation modeling technique, PLS simultaneously models the structural paths (*i.e.*, theoretical relationships among latent variables) and measurement paths (*i.e.*, relationships between a latent variable and its indicators). Rather than suppose equal weights for all indicators of a scale, the PLS algorithm allows each indicator to vary in how much it contributes to the composite score of the latent variable. Therefore, indicators with weaker relationships to related indicators and the latent construct are given lower weightings [33]. Each iteration of the algorithm introduces another latent variable. The number of latent variables was chosen to maximize cross-validated *R*^2^ (called *Q*^2^) of the training set. The model is generally considered internally predictive if *Q*^2^ > 0.5 [34], as generally the *Q*^2^ are much better indicators than standard error and conventional *R*^2^ of how reliable predictions actually are.

### 2.7. Support Vector Regression (SVR)

Recently, SVR has emerged as an alternative and powerful technique to solve regression problems by introducing an alternative loss function [35]. Such method attempts to minimize the generalization error bound by structural risk minimization (SRM) principle so as to achieve generalized performance instead of minimizing the observed training error. In this work, this method has been applied to estimate the nonlinear relationships between the OB values of molecules and their relative molecular features.

Hence, only a brief description of the method is given here. Suppose we are given training data {(*x_i_*, *y_i_*)}*_i_^n^* where *x_i_* denotes the input vector; *y_i_* denotes the output (target) value and *n* denotes the total number of data patterns. The modeling aim is to identify a regression function *y*= *f*(*x*) that accurately predicts the outputs *y_i_* corresponding to a new set of input-output examples{(*x_i_*, *y_i_*)}. Using mathematical notation, the nonlinear regression function in the original feature space is approximated using the following function:

(3) $$f(x)=w\cdot \phi (x)+b,\phi :R^{n}\to F,w\in F$$

where *w* and *b* are regression parameters. And ϕ(*x*) denotes the high-dimensional feature space, which is nonlinearly mapped from the input space. Additionally, by introducing Lagrange multipliers and exploiting the optimality constraints, the SVR function is finally formulated as following:

(4) $$f(x)=\sum_{i=1}^{n}(\alpha_{i}-\alpha_{i}^{*})K(x,x_{i})+b$$

where α *_i_* and α _i_^*^ are Lagrange multipliers, have been obtained by minimizing the regularized risk function. The kernel function *K* (*x*, *x_i_*) has been defined as a linear dot product of the nonlinear mapping, *i.e*.,

(5) $$K(x,x_{i})=\phi (x)\cdot \phi (x_{i})$$

Generally, four kinds of kernel functions, *i.e*. linear function, polynomial function, sigmoid function and radial basis function $K(x_{i},x_{j})=e^{-\frac{||x_{i}-x_{j}|{|}^{2}}{2\sigma^{2}}}$ (RBF), are available to perform prediction. Empirical studies have demonstrated that the RBF outperforms the other three kinds of kernel functions. Hence, this work adopted the RBF to perform inference process. The regularization parameter *C* and the kernel parameter *γ* were selected based on the overall accuracy of the internal five-fold cross-validation using the grid search method.

## 3. Results and Discussion

### 3.1. Dataset Division

At first, three methods including MLR, PLS and SVM were tried in order to build reasonable predictive models for OB of 805 molecules as a whole dataset. For the MLR model, its coefficients *R*^2^ for training set and testing set are 0.39 and 0.47, respectively. The PLS model with best performance has 8 latent variable, presenting *R*^2^ = 0.58 for the training set, and *Q*_ex_^2^ = 0.37 for the test set. As for the SVM model, the regression results in the poor *R*^2^ and *Q*_ex_^2^ of 0.39 and 0.35, respectively. From these results, it can be concluded that the present models generated relatively poor models for the prediction of OB values, in agreement with these reported models [8,9,11,12]. Accordingly, novel chemometric methods are needed to be introduced to improve the prediction ability for the OB of drugs.

The multidrug resistance (MDR) ATP binding cassette (ABC) proteins, especially the P-gp, are large, membrane-bound proteins, which form a functional network, capable to extrude a very wide range of foreign (xenobiotic) substrates [16]. As well as the efflux pump, the cellular route of absorption exposes drugs to intracellular metabolic systems; small intestinal enterocytes provide the first site for CYPP450-mediated metabolism of orally ingested drugs and xenobiotics [15]. For CYP3A4 and CYP2D6, the major Phase I drug-metabolizing enzymes, they are found to play complementary roles with P-gp in intestinal drug metabolism, where, through repeated extrusion and re-absorption, P-gp ensures elongated exposure of the drugs to the metabolizing enzyme [36]. Therefore, for crossing tissue barriers, in addition to some basic physical characteristics, including molecular size, charge distribution, and hydrophobicity, drug interactions with the membrane transporter P-gp and the metabolizing enzymes CYP3A4 and CYP2D6 are also key determinants [37]. Due to the critical roles of the specific membrane transport system and the intracellular metabolizing enzymes in oral drug bioavailability, it is thus reasonable to bring the information on P-gp, CYP3A4 and CYP2D6 into the creation of a new chemometric method for OB prediction.

In this work, we have divided the 805 structurally diverse drug and drug-like molecules into subsets based on the binding affinity features of the molecules with the three proteins, which significantly strengths the performance of OB models as mentioned in the following section 3.2. Here, the self-consistent method was applied to define the ranking boundary of the subsets. According to the binding features of the molecules, all compounds are iteratively used for classification analysis to ensure the optimal performance for the whole datasets. Finally, four subsets with best performance were generated: Set 1 (binding score < 5, 192 compounds), Set 2 (5 < binding score < 6, 149 compounds), Set 3 (6 < binding score < 7.5, 224 compounds) and Set 4 (binding score > 7.5, 240 compounds) (Table S1).

### 3.2. Design of Training and Test Sets

**Figure 1:**
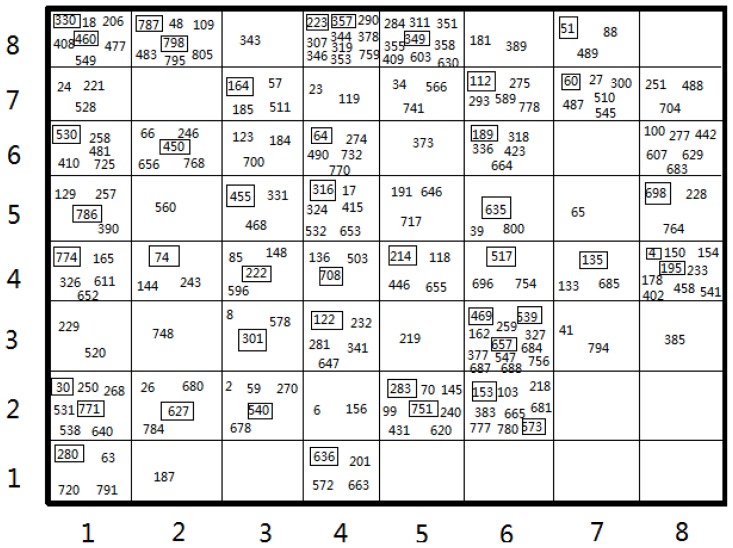
Clustering of 8 × 8 Self-organizing map (SOM) of 224 compounds in Set 3. The numbers correspond to the series numbers of the compounds. Those numbers with frames are compounds of the test set, and the others are the compounds of the training set.

The Kohonen’s self-organizing Neural Network has the special property of effectively creating a spatially organized internal representation of various features of input signals and their abstractions [31]. Therefore, the SOM method that enables to split a dataset into training and test sets assures that both sets cover the information space as good as possible. Figure 1 shows the distribution of the compounds within the best mapping in Set 3. The two-dimensional square grid presents clear division of the input pattern into 64 neurons. Our selective premise for the test set was that the test molecules distributed in the overall data set should be more representative of the overall data set and thus should lead to good predictive models [38]. Projections of the test and training set in the map indicate that these molecules are evenly distributed over the map: 54 of 64 for Set 3. For the SOM of Set 3, a relatively concentrated area appears in the middle side for the test set (numbers with frame), and several small test clusters reside in the ambient side of the map. Finally, the SOM models for Set 1, Set 2, Set 3, Set 4 were built with different data divisions of 156/36, 122/27 and 180/44 and 197/43, respectively.

### 3.3. Model Building

#### 3.3.1. The Results of MLR

**Figure 2:**
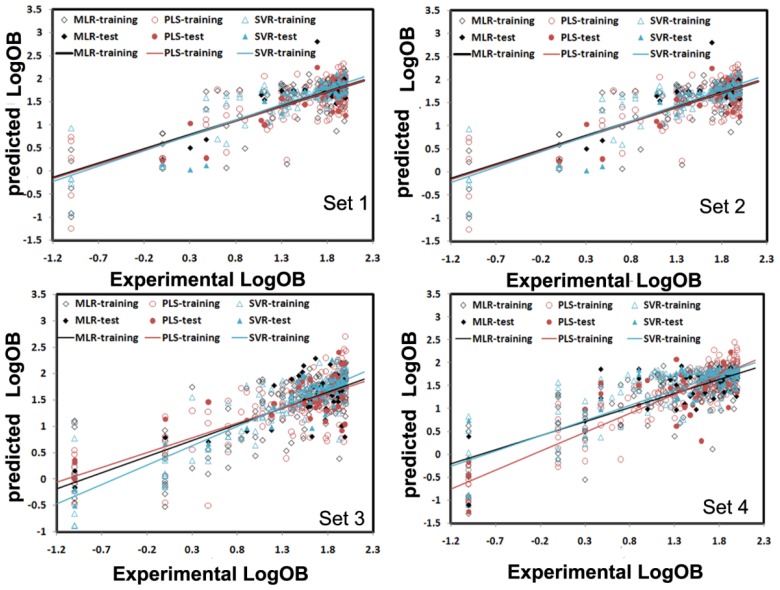
Experimental and predicted LogB values for Set 1, Set 2, Set 3 and Set 4 using the multiple linear regression (MLR), partial least squares (PLS) and support-vector machine regression (SVR) models, respectively. For MLR, the training and test sets are represented by the black empty squares and black solid squares, respectively. For PLS, they are represented by the red empty circles and red solid circles, respectively, while for SVR, they are shown by the blue empty triangles and blue solid triangles, respectively.

The MLR analysis with stepwise selection was employed to extract the molecular descriptors for creation of structure-OB relationships. The appropriate model should have reasonable *R*^2^, *F*-test, and SEE (standard error of estimation) values, but also have ability to predict the property of the test compounds (*Q*_ex_^2^, SEP, standard error of prediction) not included in the training set. The resulted correlation between experimental and predicted logB for all the compounds was shown in Figure 2.

For example, the optimal linear model was built with eleven descriptors in Set 1:

(6) $$\begin{array}{l} \text{LogB}=-16.8\;(\text{R}3\text{v}+)+8.5\;(\text{R}2\text{p}+)-2.7\;(\text{nC}=\text{O}(\text{O})2)-1.5\;(\text{nNq})-1.1\;(\text{nROCON})-\\ 0.7\;(\text{nSH})-0.2\;(\text{MATS}7\text{p})-0.1\;(\text{T }(\text{O}.\text{P}))-0.1\;(\text{O}-056)-0.04\;(\text{G }(\text{N}.\text{Br}))-\\ 0.02\;(\text{RDF}130\text{m})+1.9\\ N_{\text{tr}}=156;N_{\text{te}}=36;R^{2}=0.621;\;{Q_{\text{ex}}}^{2}=0.612;F=21.374;\text{SEE}=0.411;\text{SEP}=0.311. \end{array}$$

where *N*_tr_ and *N*_te_ are the number of compounds included in the training and test set, respectively. Predicted values from Equation (6) fell close to the experimental logB with reasonable *R*^2^ (0.6), which indicates good statistical characteristics of the model. The model’s prediction capability is further validated by the external test with rational correlation coefficients (0.612).

For the MLR equation, it is worthwhile to note that the sequential order in which these variables appeared in the OB model agreed with the order of relative contribution importance (in modulus), as derived from a subsequent standardization of the orthogonalized regression coefficients. The equation of Set 1 shows that the most important two descriptors are the GETAWAY descriptors R3v+ and R2p+. R3v+ is defined as R maximal autocorrelation of lag 3/weighted by atomic van der Waals volumes, while R2p+ is R maximal autocorrelation of lag 2/weighted by atomic polarizabilities. Since such descriptors derived from the Molecular Influence Matrix (MIM) contain local or distributed information on molecular structure, in most cases more than one GETAWAY descriptor is needed to reach an acceptable modeling power. The negative coefficient of R3v+ implies that low value of atomic van der Waals volumes can lead to increased bioavailability for a compound. While the positive coefficient of R2p+ may be interpreted as that low value of the atomic polarizabilities can lead to decreased OB of a molecule.

Hydrogen bonding interaction often plays an important role in determining the binding of a ligand-receptor. This implies that the binding of candidate drugs with proteins and metabolizing enzymes in the cellular membranes have great effects on the human bioavailability, *i.e*., the stronger binding of agents with the proteins, the more difficult the molecules overcome the cellular barriers. In fact, for the functional group counts descriptors in Equation 1, nC=O(O)2, nNq and nROCON present the E-state of hydrogen bond acceptors, while nSH is the E-state of hydrogen bond donor. Since the negative coefficients of the descriptors suggest that higher values of the factors induce lower OB value of a molecule, it is reasonable to believe that the existence of hydrogen bond acceptors and donors is actually unfavorable for the bioavailability of candidate drugs.

As for the Set 2 model, Equation 7 presents the optimal MLR model based on the eleven descriptors for the OB direction:

(7) $$\begin{array}{l} \text{LogB}=-2.3\;(\text{G}2\text{e})-1.4\;(\text{R}1\text{e})+0.8\;(\text{Mor}27\text{v})-0.8\;(\text{nCONN})+0.5\;(\text{Mor}25\text{e})-\\ 0.3\;(\text{nRCOOR})-0.4\;(\text{Mor}30\text{u})+0.3\;(\text{C}-039)+0.2\;(\text{MAXDN})+0.1\;(\text{Mor}06\text{e})-\\ 0.1\;(\text{Mor}04\text{e})+4.6\\ N_{\text{tr}}=122;N_{\text{te}}=27;R^{2}=0.521;\;{Q_{\text{ex}}}^{2}=0.542;F=10.871;\text{SEE}=0.400;\text{SEP}=0.480. \end{array}$$

The plot of experimental versus predicted logB shows that the predicted values enable to capture the experimental values with reasonable *R*^2^ value (0.521). The model’s prediction ability is also validated by the external test with correlation coefficients *Q*_ex_^2^ = 0.542 and SEP = 0.480. As the most important descriptor for the OB value in Set 2, G2e is a second component symmetry directional WHIM descriptor that involves the atomic Sanderson electronegativities as a weighting scheme [39]. It is based on the statistical indices calculated as the information content index on the symmetry along each component. The negative coefficient of the descriptor indicates that bioavailability of a candidate drug increase with decreasing molecular symmetry. For another descriptor R1e, it belongs to the same class of GETAWAY descriptors as the R3v+ and R2p+ in Set 1, which emphasizes the important roles of the GETAWAY descriptor in determining the OB values of molecules. In addition, several 3D-MoRSE descriptors are also selected to build the model, including Mor25e, Mor04e, Mor27v, Mor30u and Mor06e. They are molecule atom projections along different angles, which represent different views of the whole molecule structure. As for the functional group counts’ descriptors nCONN, nRCOOR, they represent the E-state of hydrogen bond acceptors, which are closely associated with the human bioavailability as mentioned in Equation 6.

For the MLR models of Set 3 and Set 4, their statistical characteristics are slightly worse, with lower *R*^2^ and higher residues (Table 1), and therefore detailed analysis for the results of Set 3 and Set 4 are not presented here.

#### 3.3.2. The Results of PLS

**Figure 3:**
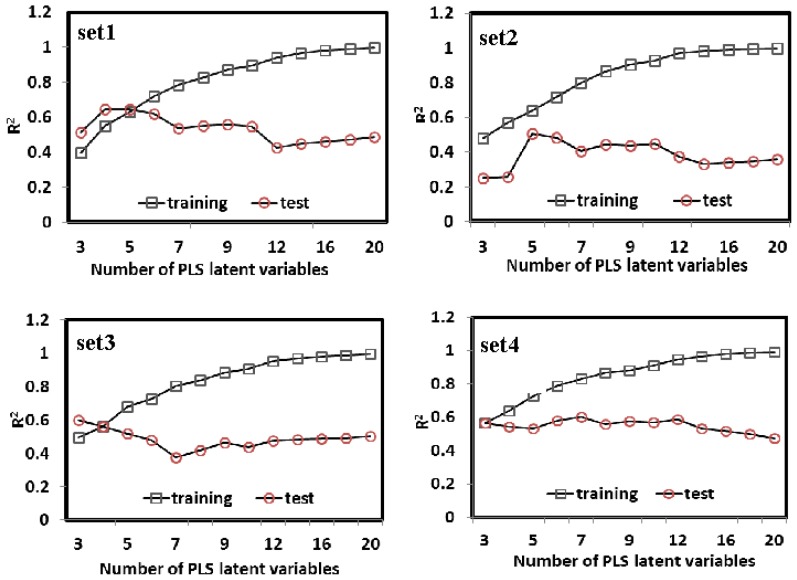
The prediction accuracies of 5-fold cross-validation for the 805 compounds derived from partial least squares analysis with latent variables varying from 3 to 20 in Set 1, Set 2, Set 3 and Set 4, respectively.

PLS is a wide class of methods for modeling relations between sets of observed variables (*Y*) by means of latent variables (orthogonal linear combinations of *X*). As a standard regression technique, PLS can handle highly correlated, noisy, and numerous *X*-variables and simultaneously predict several response variables. In this section, PLS was carried out to construct the relationships between the bioavailability of the compounds and their molecular structures, which is based on linear transformation from a large number of original descriptors to a new variable space. The numbers of the latent variables were chosen to maximize the prediction accuracy of the cross-validated dataset. As shown in Figure 3, the PLS models were built with different latent variables varying from 3 to 20 for Set 1, Set 2, Set 3 and Set 4, and finally 5, 5, 4 and 7 were selected as the optimal number of the PLS factors for each subset, respectively. Under such condition, the average error rate (cross-validation) for the 805 molecules is the smallest (~0.581) among all the cases of different number of factors. As shown in Figure 2, the obtained models are satisfactory for both the training and test sets, with no evident overfitting or over-training phenomenon. The PLS models exhibit the reasonable correlation coefficients *R*^2^ of ~0.691 with SEE of ~0.411 for the training data. All the data show that the models are externally good predictive, which indicates that PLS enables to generate relatively good models for the bioavailability of the compounds. However, compared with the MLR models, the PLS models do not display absolute advantages for the SEE, SEP, and *O*_ex_^2^ for the training and test sets.

#### 3.3.3. The Results of SVR

**Figure 4:**
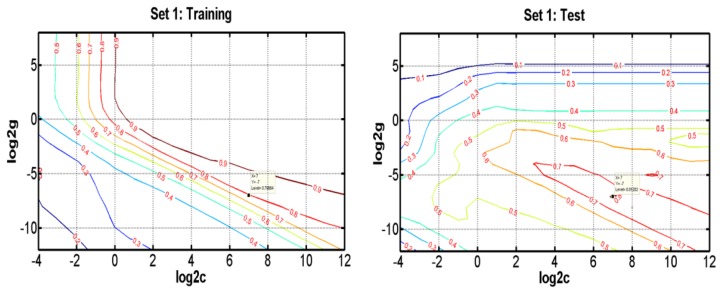

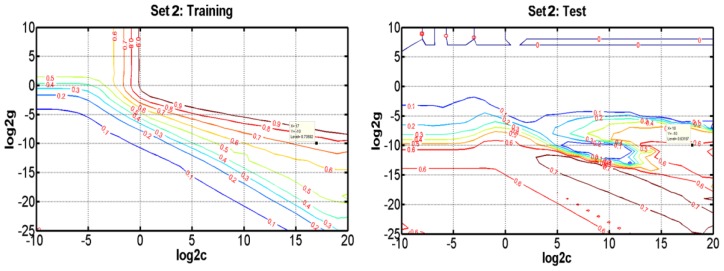
Contour plots of the optimization error for SVR when optimizing the parameters *γ* and *C* for the prediction of bioavailability for the training (**a**) and test (**b**) sets in Set 1 and Set 2.

As a new and powerful modeling tool, SVR has recently gained much interest in pattern recognition and function approximation applications. Compared with traditional regression and neural networks methods, SVRs have some advantages, including global optimum, good generalization ability, simple implementation, few free parameters, and dimensional independence [40,41]. In this study, SVR takes the most commonly used Gaussian radial basis function as the kernel function, which involves two parameters to be optimized, *i.e.*, the penalty parameter *C* and the Gaussian function parameter *γ*. To determine the optimal parameters, a grid search was performed based on leave-one-out cross validation on the training set for all parameter combinations of log2*C* ranging from −4 to 12 and log2*γ* from −12 to 8. Figure 4 and Figure S1 show the influence of each parameter with the other one fixed to the optimal values on the model performance in Set 1, Set 2, Set 3 and Set 4, respectively. The results show that the SVR models reach the best performance when *C* = 128, *γ* = 0.0078 in Set 1, *C* = 262144, *γ* = 9.77 × 10^−4^ in Set 2, *C* = 131072, *γ* = 3.05 × 10^−5^ in Set 3, and *C* = 32768, *γ* = 1.53 × 10^−5^ in Set 4. Subsequently, we selected optimal variables for SVR by varying numbers of components from 1 to 1536.

As shown in Table 1, when all the 1536 molecular descriptors were used as the input variables of SVR, the obtained regression models exhibited relatively weak *Q*_ex_^2^ of ~0.611 for the test sets despite the good determination coefficients (*R*^2^ = ~0.752) for the training sets, which reveals that the number of selected features probably have an potential effect on the prediction ability of models. Thus, the stepwise method was used to select the proper number of variables for each subset, and finally 21, 12, 18 and 12 input variables were obtained for Set 1, Set 2, Set 3 and Set 4, respectively. The resulting correlations between the experimental and predicted LogB for all the compounds were shown in Figure 3, indicating that the SVM models are superior to MLR and PLS for the OB prediction. Such results imply that the nonlinear relationship between the bioavailability and molecular structures is more notable than the linear relationship.

### 3.4. Comparison of the MLR, PLS and SVR Models

In this work, three regression methods were employed to construct reasonable predictive models for the OB values of candidate drugs. Two methods are based on the linear regression, MLR and PLS; one other method is the network SVR based on the nonlinear regression. In this work, *F*-test was applied to investigate the performance difference of the three models [42].

$$F(n_{1},n_{2})={{\text{SEP}}_{1}}^{2}/{{\text{SEP}}_{2}}^{2}$$

where, *n*_1_ and *n*_2_ are the number of samples in the test set, SEP_1_^2^ is the square from the higher and SEP_2_^2^ is the square from lower root mean square errors of the two compared models. When comparing the performances of SVR with MLR and PLS, *F*-values are 1.66, 0.56 and 0.92 for SVR/MLR in Set 1, Set 3 and Set 4, and 0.5, 0.48 and 0.88 for SVR/PLS in each subset, respectively, which are lower than the critical ones (1.74 for Set 1, 1.65 for Set 3, and 1.66 for Set 4). As for Set 2, its *F*-values are 2.57 for SVR/MLR, and 2.68 for SVR/PLS, which are much higher than the critical one (1.90). This indicates no statistically significant difference at a level of significance of 0.05 in Set 1, Set 3 and Set 4, except for Set 2. As for the two linear models, the calculated *F*-values are also lower than the critical ones for the four subsets. The results show that at a level of significance of 0.05 the differences in the performances of the mean SEP in both linear models differ only randomly. In summary, the statistical tests reveal that the performances of the MLR, PLS and SVR models are comparable with each other except for the SVR model in Set 3. This implies that the linear and non-linear methods are all appropriate for predicting the human bioavailability of candidate drugs.

For the SVR models, their good performances partly benefits from the fact that it can model nonlinear relationships between dependent and independent variables, even without prior knowledge of the form of the nonlinearity. While for the traditional neural network approaches, they have suffered difficulties with generalization, producing models that can overfit the data, which is induced by the use of optimalization algorithms used for parameter selection and the statistical measures. In addition, instead of minimizing the observed training error as the traditional methods, SVR attempts to minimize the generalization error bound so as to achieve good generalized performance. Moreover, since SVR works by solving a constrained quadratic problem where the convex objective function for minimization is given by the combination of a loss function with a regularization term, the introduction of the *ɛ* value and the regularization parameter *C* leads to better robust properties of SVR for various signal-to-noise ratios.

The prediction ability of QSAR models depends heavily on two factors, including the molecular descriptors carrying enough information of molecular structures for the interpretation of the activity/property, and the statistical method employed [43]. For the linear MLR method, features should represent the maximum information in activity variations, the minimum collinearity, and the well-understood relationships to the responses. In this work, the MLR-extracted descriptors, the R3v+, R2p+ for Set 1, G2e, R1e for Set 2, O-056, nNq for Set 3, and nArCNO, nNq for Set 4 play important roles in understanding the mechanism of human bioavailability of candidate drugs. For the MLR method, although it only allow easy model interpretation of what should be changed in a structure to improve the molecular biological activity, the prediction ability of such model obtained in this work still shows its reasonability, which indicates that the MLR method are reliable for the prediction of human bioavailability.

PLS is a useful linear technique commonly used in QSAR analysis. In this work, four conventional quantitative structure-logOB relationships were derived by the PLS analysis. As we can see from the Table 1, PLS is uniformly comparable to MLR on the datasets. The performance of PLS is slightly better that of MLR in the prediction ability and cross-validation for the Set 1 and Set 2 data. While for the Set 3 and Set 4 data, PLS performs worse than MLR in the prediction process. Compared with MLR, the advantage of PLS is that it is a bilinear modeling method in which the original *X* (predictor variables) is projected onto a small number of orthogonal latent variables (LVs) to simplify the relationship between *X* and *Y* (response variables) and mitigate the colinearity problem, which is thus relatively favorable for the prediction of bioavailability.

In summary, the results of MLR, PLS and SVR models are indicative of their abilities to accommodate linearity and nonlinearity in the bioavailability and structural descriptors. In particular, the advantages of SVR, such as robustness, no additional test requirement, and optimal prediction ability were validated in our work.

## 4. Conclusions

Automatically predicting human bioavailability is a very important issue because it helps to prevent industrial failure, human toxicity and poor drug activity. However, the application of existing OB models has been limited by the ignorance of the presence of specific membrane transport systems and intracellular metabolizing enzymes in the gastrointestinal tract. In this work, for the first time, we have constructed a novel chemometric method for prediction of human OB by integrating the information of the ATP-dependent efflux protein P-gp and the cytochrome P4503A4 and P4502D6 metabolizing enzymes, the important defence limiting the absorption of candidate drugs. To establish *in silico* models for predicting OB values of molecules, the two linear methods MLR and PLS, and the non-linear method SVR were attempted in the present work. It can be concluded from our results that the performance of MLR, PLS and SVR are all reliable, as indicated by their correlation *R*^2^ and prediction error residues. Thus, they could be helpful as complementary tools applied in “screening prior to synthesis” procedures for prediction of OB values.

## Figures and Tables

**Table 1:** Statistical results of MLR, PLS and SVR for oral bioavailability (OB) prediction of compounds.

	Set 1				Set 2				Set 3				Set 4			
	Training size		Test size		Training size		Test size		Training size		Test size		Training size		Test size	
	156		36		122		27		180		44		197		43	
	*R*^2^	SEE	*Q*_ex_^2^	SEP	*R*^2^	SEE	*Q*_ex_^2^	SEP	*R*^2^	SEE	*Q*_ex_^2^	SEP	*R*^2^	SEE	*Q*_ex_^2^	SEP
**MLR**	0.621	0.411	0.612	0.311	0.521	0.400	0.541	0.482	0.610	0.492	0.612	0.48	0.61	0.482	0.622	0.480
**PLS**	0.631	0.390	0.651	0.311	0.643	0.331	0.511	0.470	0.561	0.500	0.561	0.521	0.831	0.312	0.600	0.490
**SVM**	0.800	0.311	0.720	0.220	0.750	0.280	0.630	0.772	0.780	0.361	0.800	0.361	0.690	0.421	0.682	0.461
**SVM_T_**	0.840	-	0.731	-	0.731	-	0.310	-	0.970	-	0.590	-	0.990	-	0.561	-

*R*^2^, the regression coefficient of the training set; *Q*_ex_^2^, the regression coefficient of the test set; SEE, standard error of estimate; SEP, standard error of prediction; SVM_T_ represents the models using the total 1536 molecular descriptors as the input variables of SVR; -, not available.

## References

[1] O’Brien S.E., de Groot M.J. (2005). Greater than the sum of its parts: Combining models for useful ADMET prediction. J. Med. Chem.

[2] Beresford A.P., Selick H.E., Tarbit M.H. (2002). The emerging importance of predictive ADME simulation in drug discovery. Drug Discov. Today.

[3] Egan W.J., Merz K.M., Baldwin J.J. (2000). Prediction of drug absorption using multivariate statistics. J. Med. Chem.

[4] Chen M.L., Shah V., Patnaik R., Adams W., Hussain A., Conner D., Mehta M., Malinowski H., Lazor J., Huang S.M. (2001). Bioavailability and bioequivalence: An FDA regulatory overview. Pharm. Res.

[5] Hou T., Li Y., Zhang W., Wang J. (2009). Recent developments of *in silico* predictions of intestinal absorption and oral bioavailability. Comb. Chem. High Throughput Scr.

[6] Hou T., Wang J. (2008). Structure-ADME relationship: Still a long way to go?. Expert Opin. Drug Metab. Toxicol.

[7] Lipinski C.A., Lombardo F., Dominy B.W., Feeney P.J. (1997). Experimental and computational approaches to estimate solubility and permeability in drug discovery and development settings. Adv. Drug Deliver. Rev.

[8] Aller S.G., Yu J., Ward A., Weng Y., Chittaboina S., Zhuo R., Harrell P.M., Trinh Y.T., Zhang Q., Urbatsch I.L. (2009). Structure of P-Glycoprotein reveals a molecular basis for poly-Specific Drug Binding. Science.

[9] Yoshida F., Topliss J.G. (2000). QSAR model for drug human oral bioavailability. J. Med. Chem.

[10] Hou T.J., Wang J.M., Zhang W., Xu X.J. (2007). ADME evaluation in drug discovery. 6. Can oral bioavailability in humans be effectively predicted by simple molecular property-based rules?. J. Chem. Inf. Model.

[11] Wang Z., Yan A.X., Yuan Q.P., Gasteiger J. (2008). Explorations into modeling human oral bioavailability. Eur. J. Med. Chem.

[12] Ma C.Y., Yang S.Y., Zhang H., Xiang M.L., Huang Q., Wei Y.Q. (2008). Prediction models of human plasma protein binding rate and oral bioavailability derived by using GA-CG-SVM method. J. Pharma. Biomed.

[13] Tian S., Li Y.Y., Wang J.M., Zhang J., Hou T.J. (2011). ADME evaluation in drug discovery. 9. prediction of oral bioavailability in humans based on molecular properties and structural fingerprints. Mol. Phar.

[14] Hou T., Li Y., Zhang W., Wang J. (2009). Recent developments of *in silico* predictions of intestinal absorption and oral bioavailability. Comb. Chem. High Throughput Scr.

[15] Chan L.M., Lowes S., Hirst B.H. (2004). The ABCs of drug transport in intestine and liver: Efflux proteins limiting drug absorption and bioavailability. Eur. J. Pharm. Sci.

[16] Doherty M.M., Charman W.N. (2002). The mucosa of the small intestine: How clinically relevant as an organ of drug metabolism?. Clin. Pharmacokinet.

[17] Benet L.Z., Wu C.Y., Hebert M.F., Wacher V.J. (1996). Intestinal drug metabolism and antitransport processes: A potential paradigm shift in oral drug delivery. J. Control Rel.

[18] Borchardt R.T., Smith P., Wilson G. (1996). Models for Assessing Drug Absorption and Metabolism.

[19] Hou T.J., Xu X.J. (2002). ADME evaluation in drug discovery. 1. Applications of genetic algorithms on the prediction of blood-brain partitioning of a large set drugs from structurally derived descriptors. J. Mol. Model.

[20] Chemical Book Database.

[21] Wang X., Yang W., Xu X., Zhang H., Li Y., Wang Y.H. (2010). Studies of benzothiadiazine derivatives as hepatitis C virus NS5B polymerase inhibitors using 3D-QSAR, molecular docking and molecular dynamics. Curr. Med. Chem.

[22] Hancock T., Put R., Coomans D., vander Heyden Y., Everingham Y. (2005). A performance comparison of modern statistical techniques for molecular descriptor selection and retention prediction in chromatographic QSRR studies. Chemometr. Intell. Lab.

[23] Saíz-Urra L., González M.P., Teijeira M. (2006). QSAR studies about cytotoxicity of benzophenazines with dual inhibition toward both topoisomerases I and II: 3D-MoRSE descriptors and statistical considerations about variable selection. Bioorg. Med. Chem.

[24] Khajeh A., Modarress H. (2011). Quantitative structure–property relationship for surface tension of some common alcohols. J. Chemometr.

[25] Talete S. Dragon for windows (software for molecular descriptor calculations), version 5.4.

[26] Jain A.N. (2003). Surflex: Fully automatic flexible molecular docking using a molecular similarity-based search engine. J. Med. Chem.

[27] RCSB Protein Data Bank.

[28] Xu X., Fu J.X., Wang H., Zhang B.D., Wang X., Wang Y.H. (2011). Influence of P-glycoprotein on embryotoxicity of the antifouling biocides to sea urchin (*Strongylocentrotus intermedius*). Ecotoxicology.

[29] Xue Y., Guoyin C., Guan Y.N., Cracknell A.P., Jiakui T. (2005). Iterative self-consistent approach for Earth surface temperature determination. Int. J. Remote Sens.

[30] Vesanto J., Alhoniemi E. (2000). Clustering of the self-organizing map. IEEE Trans. Neural Networks.

[31] Wang Y., Li Y., Ding J., Wang Y., Chang Y. (2008). Prediction of binding affinity for estrogen receptor α modulators using statistical learning approaches. Mol. Divers.

[32] Höskuldsson A. (1988). PLS regression methods. J. Chemometr.

[33] Chin W.W., Marcolin B.L., Newsted P.R. (2003). A partial least squares latent variable modeling approach for measuring interaction effects: Results from a monte carlo simulation study and voice mail emotion/adoption study. Inf. Syst. Res.

[34] Wold S., Ruhe A., Wold H., Dunn W.J.J. (1984). The collinearity problem in linear regression. The partial least squares (PLS) approach to generalized inverses. Sci. Stat. Comput.

[35] Vapnik V., Golowich S., Smola A. (1997). Support Vector Method for Function Approximation, Regression Estimation, and Signal Processing. Advances in Neural Information Processing Systems 9, Proceedings of the 1996 Neural Information Processing Systems Conference NIPS 1996.

[36] Benet L.Z., Cummins C.L., Wu C.Y. (2004). Unmasking the dynamic interplay between efflux transporters and metabolic enzymes. Int. J. Pharm.

[37] Szakács G., Váradi A., Özvegy-Laczka C., Sarkadi B. (2008). The role of ABC transporters in drug absorption, distribution, metabolism, excretion and toxicity (ADME–Tox). Drug Discov. Today.

[38] Guha R., Serra J.R., Jurs P.C. (2004). Generation of QSAR sets with a self-organizing map. J. Mol. Graph. Model..

[39] Todeschini R., Gramatica P., Provenzani R., Marengo E. (1995). Weighted holistic invariant molecular descriptors. Part 2. Theory development and applications on modeling physicochemical properties of polyaromatic hydrocarbons. Chemom. Intell. Lab. Syst.

[40] Cristianini N., Shawe-Taylor J. (2000). An Introduction to Support Vector Machines.

[41] Schölkopf B., Burges C.J.C., Smola A.J. (1999). Advances in Kernel Methods: Support Vector Learning.

[42] Bhandare P., Mendelson Y., Peura R.A., Janatsch G., Kruse-Jarres J.D., Marbach R., Heise H.M. (1993). Multivariate determination of glucose in whole blood using partial least-squares and artificial neural networks based on mid-infrared spectroscopy. Appl. Spectrosc..

[43] Goodarzi M., Freitas M.P., Richard J. (2008). Feature selection and linear/nonlinear regression methods for the accurate prediction of glycogen synthase kinase-3B inhibitory activities. QSAR Comb. Sci.

